# Clinical Use of Continuous Glucose Monitoring in Critically Ill Pediatric Patients with Diabetic Ketoacidosis

**DOI:** 10.1089/dia.2023.0012

**Published:** 2023-07-31

**Authors:** Esther Park, Minsun Kim

**Affiliations:** ^1^ Department of Pediatrics, Jeonbuk National University Children's Hospital, Jeonju, Korea.; ^2^ Research Institute of Clinical Medicine of Jeonbuk National University-Biomedical Research Institute of Jeonbuk National University Hospital, Jeonju-si, Korea.; ^3^ Department of Pediatrics, Jeonbuk National University Medical School, Jeonju-si, Korea.

**Keywords:** Continuous glucose monitoring, Diabetic ketoacidosis, Pediatric intensive care unit

## Abstract

**Background:**

The use of continuous glucose monitoring (CGM) in pediatric patients with diabetic ketoacidosis (DKA) remains investigational, and data on its accuracy in pediatric intensive care units (PICU) are limited. This study evaluated the accuracy of three CGM devices in pediatric patients with DKA in the PICU.

**Methods:**

We compared 399 matched pairs of CGM and point-of-care capillary glucose (POC) values and grouped patients based on whether they changed their CGM sensor during their PICU stay.

**Results:**

Eighteen patients with a mean age of 10.98 ± 4.20 years were included, with three patients in the sensor change group. The overall mean absolute relative difference (MARD) was 13.02%. The Medtronic Guardian Sensor 3 (*n* = 331), Dexcom G6 (*n* = 41), and Abbott FreeStyle Libre 1 (*n* = 27) showed MARD values of 13.40%, 11.12%, and 11.33%, respectively. The surveillance error grid (SEG), Bland–Altman plot, and Pearson's correlation coefficient demonstrated satisfactory clinical accuracy of the CGM devices (SEG zones A and B, 98.5%; mean difference, 15.5 mg/dL; Pearson's correlation coefficient [*r*^2^], 0.76, *P* < 0.0001). MARD was significantly lower in subjects who did not experience a sensor change (11.74% vs. 17.31%, *P* = 0.048). Also, a statistically significant negative correlation was found between serum bicarbonate levels and POC–CGM values (*r* = −0.34, *P* < 0.001).

**Conclusions:**

The severity of DKA has a major effect on reducing the accuracy of the CGM, especially during the first several days in the intensive care unit. The reduced accuracy appears to be related to acidosis, as reflected in the serum bicarbonate levels.

## Introduction

Continuous glucose monitoring (CGM) systems offer valuable glucose data for patients with diabetes mellitus.^1,2^ It is generally used in the outpatient setting.^2^ Use of CGM devices has recently increased in hospital and intensive care unit (ICU) settings, especially during the coronavirus disease 2019 (COVID-19) pandemic, to help minimize infectious exposures.^2–4^

Diabetic ketoacidosis (DKA) is a life-threatening complication in patients with uncontrolled diabetes mellitus.^5–7^ In population studies, pediatric mortality rates range from 0.15% to 0.30%.^7^ Critically ill children should be treated in a pediatric ICU (PICU).^6,7^ Point-of-care capillary glucose (POC) measurement is the standard glucose testing method for hospitalized patients with diabetes mellitus.^8^ It is recommended that POC glucose values be obtained hourly during for patients with DKA.^6,7^ Unfortunately, frequent fingerstick (capillary blood) glucose measurements result in patient discomfort and increased nursing workload, particularly in pediatric patients and during the COVID-19 pandemic.^9,10^

CGM is a minimally invasive device that provides interstitial glucose levels without requiring repetitive fingerstick testing and this can be expected to be a helpful in the management of DKA.^11,12^ The use of a CGM device in pediatric patients remains investigational, with only very modest of data on its accuracy and feasibility in the PICU setting.^13–15^

We evaluated the accuracy and feasibility of CGM devices in pediatric patients with DKA in a PICU setting. We analyzed the accuracy of CGMs device compared to POC in 18 critically ill hospitalized pediatric patients with DKA, and also investigated the effects of patients' clinical status and laboratory data on the accuracy of the CGM.

## Methods

### Study design

A retrospective chart review was performed to collect data from the medical records of pediatric patients aged <19 years who were admitted to the PICU with DKA between July 2019 and June 2022 at Jeonbuk National University Children's Hospital (Jeonju, South Korea). Twenty-seven pediatric patients had CGM device sensors inserted during their PICU admission. When patients with diabetes, including DKA, were admitted to the PICU, we explained the usefulness of CGM devices to the parent or guardian as a part of the routine clinical practice in our hospital. If the parent agreed to participate, the guardian selected one of three different CGM devices available at our hospital, and usage was initiated. Diagnosis of DKA was based on the guidelines of the International Society for Pediatric and Adolescent Diabetes 2018.^7^

DKA severity was categorized based on the degree of acidosis at admission; mild (pH <7.3 or bicarbonate <15 mmol/L), moderate (pH <7.2 or bicarbonate <10 mmol/L), and severe (pH <7.1 or bicarbonate <5 mmol/L).^7^ Eighteen patients were enrolled in this study: we excluded one patient with more than a 10-day length of stay in the PICU and eight patients without simultaneous usage of CGM and POC. Patients were grouped based on a history of CGM sensor changes for any reason while in the PICU. The study was approved by the Institutional Review Board (IRB) of Jeonbuk National University Hospital (IRB No. 2022-10-026). No informed consent was required because the data were collected via a retrospective chart review and no personally identifiable information was shown.

### Glucose monitoring system

Three different CGM systems, the Medtronic Guardian Sensor 3 (11 patients), the Dexcom G6 (6 patients), and the Abbott FreeStyle Libre 1 (1 patient) were used to monitor the interstitial glucose levels. A CGM sensor wire was placed on the lower abdomen. The CGM reading range for the Medtronic Guardian Sensor 3 and Dexcom G6 is 40–400 mg/dL, and for the Abbott FreeStyle Libre 1 is 40–500 mg/dL.^3^ The Medtronic Guardian Sensor 3 requires calibration every 12 h or when the calibration alarm is displayed.^16^ Calibration best practice involves calibration three or four times a day.^16^

Unlike the Medtronic Guardian Sensor 3, the Dexcom G6 and Abbot FreeStyle Libre 1 are calibrated at the factory during the manufacturing process, so periodic calibration using through self-glucose measurement is not required.^17^ Reference glucose values were obtained from POC testing (Accu-Chek Inform II; Roche Diagnostics). Accu-Chek Inform II uses a mutant variant of quinoprotein glucose dehydrogenase chemistry.^18^ Tran and colleagues evaluated the accuracy of the Accu-Chek Inform II compared with the perchloric acid hexokinase comparator method on the Cobas 6000 analyzer in critically ill patients; Accu-Chek Inform II showed 97%–98% of results within ±15 mg/dL.^19^ Except for the calibration data, the CGM values were paired with the closest POC glucose values within 5 min to calculate apparent errors and mean absolute relative difference (MARD).

### Measures and outcomes

Data was obtained from electronic medical records. The following clinical data were collected: age, sex, height, body weight, body mass index (BMI), ICU length of stay, hospital length of stay, type of diabetes, occurrence of new-onset diabetes, CGM initiation time with regard to ICU admission, degree of body weight loss, and Glasgow coma scale. BMI, height, and body weight were converted into standard deviation scores (SDS) using the 2017 Korean National Growth Charts.^20^ Laboratory data, including pH, bicarbonate, base excess, anion gap, and lactate levels, were measured using blood gas analyzer. Serum electrolyte level, blood urea nitrogen, creatinine, white blood cell count, hematocrit, platelet count, C-reactive protein, HbA1c, serum C-peptide level, and urine ketone levels were measured per request of the managing health care providers.

The estimated glomerular filtration rate (eGFR) was calculated using the updated Schwartz equation.^21^ The primary outcome was the accuracy of the CGM device in pediatric patients with DKA in the PICU setting, and the secondary outcomes were the patients' clinical and laboratory data which might affect the accuracy of the CGM.

### Statistical analysis

All statistical analyses were performed using the IBM SPSS software for Windows (version 20; IBM Corp., Armonk, NY). The accuracy of the CGM device was evaluated using the surveillance error grid (SEG),^22^ Bland–Altman (BA) plot,^23^ and Pearson's correlation coefficient analyses. SEG analysis was used to assess the degree of clinical risk from inaccurate data obtained from the CGM devices.^22^ BA analysis was used to evaluate the bias in the mean difference.^23^ The Pearson's correlation coefficient (*r*^2^) was calculated to test the accuracy of the CGM device and the associations between laboratory markers and different values of POC and CGM values (POC–CGM values).

We also calculated the MARD to assess the accuracy.^24^ The MARD expressed as a percentage is the mean absolute difference between the reference (Accu-Chek Inform II) and CGM measurements; smaller MARD values indicate better accuracy.^25^ We analyzed the daily MARD and blood gas analyses by duration of stay in the PICU. The Jonckheere–Terpstra test was used to analyze trends for continuous variables by duration of stay in the PICU. Groups were compared using the Mann-Whitney U test and Kruskal–Wallis test for continuous variables, and Fisher's exact test for categorical variables. Data are presented as mean ± standard deviation for continuous variables and count (%) for categorical variables. The MARD values are presented as mean ± standard error of the mean. Statistical significance was set at *P* < 0.05.

## Results

Eighteen pediatric patients hospitalized for DKA who underwent CGM device insertion in the PICU were included. Clinical characteristics of the patients are presented in Table 1. Mean age of patients was 10.98 ± 4.20 years (range, 2–18), and four (22.2%) were male. The mean height and weight were 0.50 ± 0.99 and 0.27 ± 1.46 SDS, respectively, and the mean BMI was 0.08 ± 1.50 SDS. There were 15 patients with type 1 diabetes and 3 with type 2 diabetes. Fifteen patients were newly diagnosed with diabetes—12 with type 1 diabetes, and 3 with type 2 diabetes. HbA1c and mean glucose at admission were 13.30% ± 2.38% and 498.72 ± 172.88 mg/dL, respectively.

**Table 1.** Clinical Characteristics of Patients in the Two Groups

Clinical characteristics	All	Sensor nonchange group	Sensor change group	*P*
*N* (%)	18	15 (83.3)	3 (16.7)	
Sex, male (%)	4 (22.2)	3 (20.0)	1 (33.3)	1.000
Age (years)	10.98 ± 4.20	10.78 ± 4.50	12.00 ± 2.59	0.654
Height (SDS)	0.50 ± 0.99	0.43 ± 1.01	0.83 ± 1.01	0.912
Weight (SDS)	0.27 ± 1.46	0.33 ± 1.52	0.01 ± 1.36	1.000
BMI (SDS)	0.08 ± 1.50	0.10 ± 1.56	−0.03 ± 1.43	0.912
Diagnosis				0.442
Type 1 diabetes	15 (83.3)	13 (86.7)	2 (66.7)	
Type 2 diabetes	3 (16.7)	2 (13.3)	1 (33.3)	
New-onset diabetes	14.28 ± 1.36	14.13 ± 1.46	15.00 ± 0.00	0.301
Severity of DKA				0.375
Mild	5 (27.8)	5 (33.3)	0 (0.0)	
Moderate	3 (16.7)	3 (20.0)	0 (0.0)	
Severe	10 (55.5)	7 (46.7)	3 (100.0)	
Weight loss (%)	10.12 ± 4.41	9.44 ± 4.16	13.53 ± 4.80	0.164
Glasgow Coma Scale at admission	14.28 ± 1.36	14.13 ± 1.46	15.00 ± 0.00	0.301
Duration of insulin infusion (h)	38.15 ± 20.33	36.03 ± 21.64	46.62 ± 13.53	0.248
ICU time for CGM initiation (h)	20.84 ± 18.87	22.76 ± 20.08	11.25 ± 6.38	0.407
ICU length of stay (days)	3.81 ± 1.51	3.58 ± 1.55	4.94 ± 0.67	0.139
Hospital length of stay (days)	15.11 ± 3.34	15.00 ± 3.55	15.67 ± 2.52	0.738
CGM system				0.596
Medtronic	11 (61.1)	8 (53.3)	3 (100.0)	
Dexcom	6 (33.3)	6 (40.0)	0 (0.0)	
FreeStyle Libre	1 (5.6)	1 (6.7)	0 (0.0)	
Initial laboratory data				
pH	7.11 ± 0.15	7.13 ± 0.15	7.03 ± 0.13	0.426
Bicarbonate (mmol/L)	7.14 ± 6.90	8.32 ± 6.94	1.23 ± 2.14	0.076
Random glucose (mg/dL)	498.72 ± 172.88	485.80 ± 185.73	563.33 ± 71.18	0.250
HbA1c (%)	13.30 ± 2.38	13.16 ± 2.57	14.00 ± 0.96	0.738
Corrected sodium (mmol/L)	138.49 ± 4.08	137.77 ± 4.00	142.08 ± 2.62	0.076
Potassium (mmol/L)	4.25 ± 0.80	4.29 ± 0.79	4.07 ± 1.05	0.738
Anion gap (mL/dL	23.38 ± 5.42	22.45 ± 5.34	28.03 ± 3.41	0.130
Base excess (mmol/L)	−20.80 ± 8.85	−19.39 ± 8.96	−27.87 ± 3.70	0.164
Lactate (mmol/L)	1.84 ± 1.08	1.95 ± 1.13	1.30 ± 0.62	0.426
BUN (mg/dL)	17.44 ± 17.53	17.47 ± 19.30	17.33 ± 2.30	0.100
Creatinine (mg/dL)	0.93 ± 0.28	0.89 ± 0.29	1.13 ± 0.18	0.195
eGFR (mL/min/1.73 m^2^)	68.26 ± 20.57	70.71 ± 21.44	55.99 ± 10.25	0.203
C-peptide (serum, ng/mL^a^)	0.61 ± 0.40	0.55 ± 0.37	0.83 ± 0.60	0.500
Ketone (urine dipstick)	3.78 ± 0.55	3.73 ± 0.59	4.00 ± 0.00	0.654
WBC (10^3^/ul)	13.46 ± 9.11	12.75 ± 9.76	17.04 ± 4.06	0.130
Hematocrit (%)	45.77 ± 5.67	46.25 ± 5.92	43.37 ± 4.2	0.250
Platelet (10^3^/uL)	338.40 ± 111.53	327.40 ± 101.42	393.67 ± 168.05	0.654
CRP (mg/L^b^)	4.93 + 7.25	5.56 + 7.89 (13)	2.23 + 2.67 (3)	1.000
POC glucose (mg/dL) at the time of sensor placement	225.61 ± 59.06	233.20 ± 60.08	187.66 ± 42.34	0.301
On first day of sensor wear				
pH		7.29 ± 0.10	7.15 ± 0.07	<0.001
Bicarbonate (mmol/L)		13.91 ± 5.11	5.31 ± 3.07	<0.001
Anion gap (mL/dL)		11.67 ± 6.02	19.70 ± 4.66	<0.001
Base excess (mmol/L)		−11.75 ± 6.63	−23.07 ± 5.10	<0.001

Data are presented as the mean ± SD or number of patients (%).

The items marked with small letters represent the total number of items as follows: ^a^C-peptide (serum; *n* = 9; sensor nonchange group, *n* = 7; sensor change group, *n* = 2) and ^b^CRP (*n* = 16; sensor nonchange group, *n* = 13; sensor change group, *n* = 3).

BMI, body mass index; BUN, blood urea nitrogen; CGM, continuous glucose monitoring; CRP, C-reactive protein; DKA, diabetic ketoacidosis; eGFR, estimated glomerular filtration rate; ICU, intensive care unit; POC, point-of-care capillary glucose; SD, standard deviation; SDS, standard deviation score; WBC, white blood cell.

The average total duration of continuous intravenous insulin infusion was 38.15 ± 20.33 h. CGM initiation occurred at 20.84 ± 18.87 h after admission to the PICU. The PICU length of stay was 3.81 ± 1.51 days, and the hospital length of stay was 15.11 ± 3.34 days. None of the patients required mechanical ventilation, continuous renal replacement therapy, vasopressors, or inotropic treatment. The CGM device sensors had no sensor dislodgment and there were no adverse skin reactions or infections. Fifteen and three patients were identified in the sensor nonchange and sensor change groups, respectively. Three patients with the Medtronic Guardian Sensor 3 required sensor changes during their ICU stay because of calibration errors. The baseline characteristics and initial laboratory data showed no statistically significant differences between the groups with and without change of sensors during the PICU stay.

On the first day of CGM sensor wear, the sensor change group showed decreased levels of pH, bicarbonate, and base excess (pH, 7.29 ± 0.10 vs. 7.15 ± 0.07; bicarbonate, 13.91 ± 5.11 vs. 5.31 ± 3.07 mmol/L; base excess, −11.75 ± 6.63 vs. −23.07 ± 5.10 mmol/L, all *P* < 0.001). The anion gap increased in the sensor change group (11.67 ± 6.02 vs. 19.70 ± 4.66 mL/dL, *P* < 0.001).

**FIG. 1.**
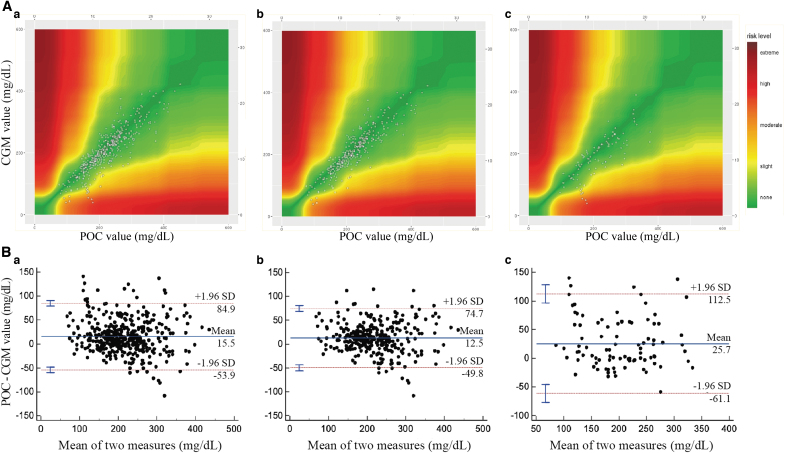
**(A)** Surveillance error grid analysis for all comparison points between CGM and POC values. **(A.a)** overall, **(A.b)** sensor nonchange group, **(A.c)** sensor change group. **(B)** Bland–Altman plot analysis showing the distribution of the difference between POC and CGM in the function of mean glucose **(B.a)** overall, **(B.b)** sensor nonchange group, **(B.c)** sensor change group. CGM, continuous glucose monitoring; POC, point-of-care capillary glucose.

Three hundred ninety-nine matched pairs of CGM and POC values were analyzed (307 pairs in the sensor nonchange group and 92 pairs in the sensor change group). All CGM device sensors were inserted after the POC level decreased to <400 mg/dL. The overall mean and range of POC levels were 234 and 76–451 mg/dL, respectively. One hundred eight pairs (27.07%) were in the range of 70–180 mg/dL, and 291 pairs (72.93%) were in the range of >180 mg/dL of POC. There were no episodes of POC hypoglycemia (<70 mg/dL). In the overall SEG analysis, 98.5% of readings were in the “none” or “slight” risk zones (Fig. 1A).

In the sensor nonchange group, 99.3% of readings were in the “none” or “slight” risk zones, a small percentage (95.7% of glucose readings) in the sensor change group were in the “none” or “slight” risk zones. With BA analysis, the mean difference between POC and CGM value was 15.5 mg/dL, and the limits of agreement ranged from −53.9 to 84.9 mg/dL (Fig. 1B). In the “sensor nonchange” and “sensor change” groups, the mean differences between POC and CGM value were 12.5 and 25.7 mg/dL, respectively; the limits of agreement ranged from −49.8 to 74.7 mg/dL and −61.1 to 112.5 mg/dL, and the Pearson's correlation coefficients (*r*^2^) were 0.81 and 0.61, respectively (*P* < 0.0001), and the Pearson's correlation coefficient (*r*^2^) for all paired measurements was 0.76 (*P* < 0.0001) (Table 2).

**Table 2.** Summary of the Accuracy of the Continuous Glucose Monitoring Devices in the Two Groups

	All	Sensor nonchange group	Sensor change group	*P* ^a^
Overall, *n* (matched pairs)	399	307	92	
*r*, *r*^2^ (*P* value)	0.87, 0.76 (0.000)	0.90, 0.81 (0.000)	0.78, 0.61 (0.000)	
MARD (%)	13.02 ± 0.63	11.74 ± 0.60	17.31 ± 1.79	0.048
Glucose level 70–180 mg/dL, *n*	108	75	33	
MARD (%)	17.56 ± 1.67	14.57 ± 1.59	24.37 ± 3.89	0.039
Glucose level >180 mg/dL, *n*	291	232	59	
MARD (%)	11.34 ± 0.57	10.83 ± 0.59	13.36 ± 1.55	0.490

Data are presented as mean ± SEM for MARD.

^a^ Mann–Whitney U tests were used to compare groups with and without a history of sensor changes.

MARD, mean absolute relative difference; *r*, *r*^2^, Pearson's correlation coefficient; SEM, standard error of the mean.

We also observed an overall MARD of 13.02% ± 0.63%. The MARD was 17.56% ± 1.67% in euglycemia (70–180 mg/dL), and 11.34% ± 0.57% in hyperglycemia (>180 mg/dL). The MARD was statistically significantly smaller in the sensor nonchange group (11.74% ± 0.60% vs. 17.31% ± 1.79%, *P* = 0.048). During the PICU stay, we observed a statistically significant tendency to decrease in MARD as duration of stay increased from the day-to-day analysis (Table 3). The bicarbonate, pH, and base excess all tended to increase significantly as the PICU length of stay increased. The MARD on the fifth day of ICU stay was 8.79% ± 0.87%. A significant negative correlation was found between serum bicarbonate levels and POC–CGM values, which was more evident in the sensor change group (all, *r* = −0.341, *P* < 0.001; sensor change group, *r* = −0.476, *P* = 0.012) (Table 4).

**Table 3.** Changes in Laboratory Markers by Intensive Care Unit Day

PICU day	1	2	3	4	5	6	7	*P* ^a^	*P* ^b^	*z *value*^b^*
pH	7.21 ± 0.12	7.29 ± 0.08	7.39 ± 0.06	7.43 ± 0.39	7.42 ± 0.04	7.42 ± 0.47	7.40 ± 0.10	<0.001	<0.001	12.227
Bicarbonate (mmol/L)	9.27 ± 5.31	14.00 ± 5.27	19.49 ± 3.69	23.10 ± 2.05	23.59 ± 2.57	24.22 ± 2.31	25.90 ± 3.39	<0.001	<0.001	13.626
Base excess (mmol/L)	−17.99 ± 7.69	−11.90 ± 5.56	−4.74 ± 4.16	−0.70 ± 2.24	−0.34 ± 2.75	0.28 ± 2.72	1.55 ± 4.45	<0.001	<0.001	13.844
Overall, *n* (matched pairs)	28	102	105	74	54	29	7			
MARD (%)	18.80 ± 2.60	15.38 ± 1.25	12.70 ± 1.51	13.11 ± 1.29	8.79 ± 0.87	8.73 ± 1.38	9.94 ± 3.26	0.001	<0.001	−3.906
Sensor non-change group, *n*	28	82	80	57	39	16	5			
MARD (%)	18.80 ± 2.60	13.77 ± 1.34	9.49 ± 0.98	11.44 ± 1.23	8.19 ± 0.96	10.33 ± 1.95	10.59 ± 4.67			
Sensor change group, *n*	0	20	25	17	15	13	2			
MARD (%)		22.00 ± 2.83	22.97 ± 5.06	18.70 ± 3.55	10.36 ± 1.89	6.77 ± 1.84	8.32 ± 0.05			
*P* value^c^		0.004	0.155	0.134	0.339	0.148	0.699			

Data are presented as mean ± SD for pH, bicarbonate, and base excess. Data are presented as mean ± SEM for MARD values.

^a^ Kruskal–Wallis tests were used to compare pH, bicarbonate, base excess, and MARD according to ICU day.

^b^ The Jonckheere–Terpstra test was used to analyze trends for continuous variables according to PICU admission.

^c^ Mann-Whitney U tests were used to compare groups with and without a history of sensor changes in MARD of each PICU day.

PICU, pediatric intensive care unit.

**Table 4.** Linear Correlations Between Laboratory Markers

	All	Sensor nonchange group	Sensor change group
POC–CGM value, *r* (*P* value)			
Bicarbonate	−0.341 (<0.001)	−0.295 (0.002)	−0.476 (0.012)
Base excess	−0.329 (<0.001)	−0.285 (0.002)	−0.467 (0.013)
MARD, *r* (*P* value)			
Bicarbonate			−0.388 (0.045)
Base excess			−0.399 (0.044)

POC, point-of-care capillary glucose.

Interstitial glucose levels were monitored using three different CGM systems: the Medtronic Guardian Sensor 3 (*N* = 11, 331 pairs), the Dexcom G6 (*N* = 6, 41 pairs), and the Abbott FreeStyle Libre 1 (*N* = 1, 27 pairs). The MARD values for Medtronic, Dexcom, and Libre were 13.40%, 11.12%, and 11.33%, respectively, but there was no significant difference, and for the Freestyle Libre with only one sensor, the MARD value is not sufficiently well determined (with *N* = 1) to make comparisons with the other two types of sensor (Table 5). The MARD value by the day of CGM sensor wear was also analyzed, and there was no significant difference in the MARD value between ICU and sensor wear days (Table 6).

**Table 5.** Summary of the Accuracy of the Continuous Glucose Monitoring Devices According to the Continuous Glucose Monitoring System

	Medtronic Guardian Sensor 3	Dexcom G6	Abbott FreeStyle Libre 1	*P* ^a^
Overall, *n* (matched pairs)	331	41	27	
*r*, *r*^2^ (*P* value)	0.86, 0.74 (0.000)	0.91, 0.83 (0.000)	0.97, 0.94 (0.000)	
MARD (%)	13.40 ± 0.73	11.12 ± 1.35	11.33 ± 1.03	0.739
Glucose level 70–180 mg/dL, *n*	87	21	0	
MARD (%)	19.95 ± 1.99	11.82 ± 1.96		0.249
Glucose level >180 mg/dL, *n*	244	20	27	
MARD (%)	11.42 ± 0.65	10.39 ± 1.87	11.33 ± 1.03	0.877

Data are presented as mean ± SEM for MARD values.

^a^ Mann–Whitney U tests were used to compare the Medtronic sensor and Dexcom devices.

**Table 6.** Comparison of Mean Absolute Relative Differences Between Intensive Care Unit and Sensor Wear Days

Days	MARD of ICU day (*n*)	MARD of sensor day (*n*)	*P* ^a^
1	18.80 ± 2.60 (28)	17.29 ± 1.50 (64)	0.799
2	15.38 ± 1.25 (102)	15.05 ± 1.26 (143)	0.389
3	12.70 ± 1.51 (105)	11.32 ± 1.26 (85)	0.692
4	13.11 ± 1.29 (74)	9.51 ± 1.10 (50)	0.156
5	8.79 ± 0.87 (54)	8.37 ± 1.20 (35)	0.650
6	8.73 ± 1.38 (29)	8.83 ± 1.70 (18)	0.768
7	9.94 ± 3.26 (7)	12.27 ± 5.63 (4)	0.788

Data are presented as mean ± SEM.

^a^ Mann–Whitney U tests were used to compare the ICU and sensor day.

*n*, matched pairs.

## Discussion

Our findings showed that the CGM device was safe and clinically effective tool for CGM in critically ill pediatric patients during PICU admission for DKA treatment. Our study reported the most improved MARD compared to previous studies on DKA treatment. In our study, all patients tolerated the CGM sensors well, without adverse effects such as sensor dislodgment, skin reaction, or infection. We believe that this may be the first study to show that marked changes in serum bicarbonate levels may affect CGM accuracy.

Patients with DKA often have rapid changes in their blood glucose levels because of impaired homeostatic mechanisms and therapeutic interventions such as insulin or dextrose.^26^ Therefore, POC levels should be monitored hourly.^6,7^ However, frequent fingerstick tests can increase patient discomfort and nursing workload.^9^ Therefore, a CGM device may be a helpful tool for detecting changing glucose trends in the PICU setting for patients with DKA who require continuous intravenous insulin infusion and frequent fingerstick tests.

CGM provides interstitial glucose levels and the direction and magnitude of the glucose trend.^8^ A CGM device has advantages over POC glucose testing, including reducing hypoglycemic and hyperglycemic episodes and the frequency of fingersticks.^3,4,8^ Use of CGM makes it possible to reduce the need for use of personal protective equipment and mitigation of the exposure risk for health care staff.^3,4^ CGM devices have been used in various pediatric clinical settings, including critical care.^12^ However, currently they are only rarely used for therapeutic decisions in the PICU setting, particularly for patients with DKA.

Previous studies have evaluated the accuracy of CGM devices during treatment of DKA.^10,13,27,28^ Gangu et al.^13^ evaluated the accuracy of a CGM device compared with POC in 12 pediatric patients with DKA (age range, 7–17 years) admitted to the PICU. A total of 103 paired measurements estimated that MARD was 20%, and the correlation of POC and CGM values was 0.682 (*P* < 0.0001).^13^ The Clark error grid showed 96% of observation in zones A and B.^13^ Jacobs et al.^10^ evaluated the accuracy of a CGM device, the Medtronic Guardian, compared with POC in 29 adult patients, including 18 patients with DKA in the ICU setting. They reported a MARD of 17.4%, and linear regression coefficient of 0.834 (*P* < 0.0001).^10^

Bichard et al.^28^ performed a feasibility study comparing a CGM device, the FreeStyle Libre Pro system, and POC in ten patients aged between 18 and 75 years with DKA. The mean POC value (200.0 mg/dL; range, 75.0–340.5 mg/dL) was higher than the mean CGM value (165.8 mg/dL; range, 46.8–324.3 mg/dL) in the 167 matched pairs, and the correlation was 0.84 (*P* < 0.001).^28^ Pott et al.^27^ compared the accuracy of a CGM device, the Dexcom G6, to POC value in 35 pediatric patients with DKA (age, 11.9 ± 4.1 years). The Clark error grid of CGM versus POC revealed 95.4% of observations within zones A and B.^27^

Our calculated overall MARD of 13.02% ± 0.63% showed the most improved value compared to the previously reported MARD in patients with DKA, even though we included three patients with sensor replacements.^10,13^ The better accuracy of the CGM device in our study may be due, in part, to the larger sample size than that used in other studies on DKA or due to the arbitrary choice of sensor types. On the fifth day of PICU stay, the MARD fell to 8.79% ± 0.87%. This is similar to the recently reported average MARD for multiple subjects of the Dexcom G6 system.^17^ MARD on the fifth day of DKA treatment appeared to be roughly comparable to that in the outpatient setting. SEG analysis also indicates that the CGM values had an almost clinically significant effect on therapeutic decisions. CGM devices may be used for clinically appropriate management of DKA.

The factors affecting MARD values can be divided into those of intrinsic performance of the CGM system and not CGM system-inherent factors.^24^ CGM system-inherent factors include calibration, stability of the CGM sensor over time, sensor-to-sensor variation, and the algorithms and smoothing filters for signal processing in the CGM system.^24^ Non-CGM system-inherent factors include insertion factors, physiologic lag time, range of the paired glucose values, and rate of change in blood glucose.^24^ Few studies have investigated factors affecting the accuracy of CGM devices in the ICU setting. Pott et al.^27^ reported that the serum bicarbonate level did not affect the accuracy of a CGM device in pediatric patients with DKA. Piper et al.^15^ evaluated the correlation between sensor performance and clinical findings in children during and after cardiac surgery. They reported no effect of body temperature, inotrope dose, or edema on the accuracy of a CGM device.^15^

Branco et al.^14^ analyzed the factors influencing the accuracy of a CGM device in 14 children requiring mechanical ventilation with at least 2 organ system failures, in the absence of diabetes. Multiple regression analysis revealed that POC, CGM, and base deficits were associated with MARD.^14^ Branco et al.^14^ first reported factors affecting MARD values but there were no significant linear correlations between the MARD and axillary temperature, vasopressor dose, lactate level, or base deficit.^14^ Previous studies have not investigated the accuracy of CGM devices in relationship to pH, serum bicarbonate, anion gap, and laboratory tests. However, our study—evaluating 138 pairs of data—showed negative correlation between POC–CGM values and serum bicarbonate levels. Lower serum bicarbonate levels may affect the accuracy of CGM, such as intrinsic sensor performance or physiologic lag time.

Lag time is affected by several physiological factors, such as the local blood flow, the tissue perfusion, and the interstitial fluid's permeability.^29^ Acidosis in DKA is often associated with dehydration and tissue hypoperfusion; therefore, lower serum bicarbonate levels may be related to prolonged lag time. This implies that, as serum bicarbonate levels improve, the accuracy of the CGM device may also improve. This may also explain our result; MARD improved when DKA was improved during the PICU stay.

During our evaluation, three patients required more than one sensor owing to calibration errors. A calibration error alarm was triggered when the reference glucose value used for calibration differed significantly from the CGM values. This error indicates that the correlation between the CGM and reference glucose is very poor. On the first day of sensor wear, serum bicarbonate, pH, and base excess were significantly lower in the sensor-change group than in the nonchange group. These results may also affect accuracy of CGM.

Our study had some limitations. First, sample size was 18 patients and 399 paired samples which may limit the interpretation of the results. However, the CGM devices had a good correlation with POC values and MARD compared to previous studies of CGM use to monitor patients with DKA treatment. Second, the timing of CGM device sensor insertion varies from patient to patient. Our study's mean PICU stay of CGM sensor insertion was 20.84 ± 18.87 h. Within 24 h after PICU admission, 14 of 18 patients underwent CGM device sensor insertion. Only 28 paired values were recorded on the first day of the PICU stay. Also, the number of paired data measurements by the ICU days were not consistent. Finally, the patients in our study did not use the same CGM device.

In our study, the measured CGM range was wide, between 40 and 422 mg/dL, and the POC range was 76 and 451 mg/dL. The strength of our study is that it enables us to evaluate the accuracy of the CGM device for a wide range of glucose values.

Our results suggest that the CGM devices can help assess glucose levels during DKA treatment in the PICU setting. We hope to use the CGM device instead of hourly POC glucose monitoring in patients with DKA. Further bigger better studies are needed using newer crop of CGM devices, with more patients and more pairs of observations.
